# The position of geochemical variables as causal co-factors of diseases of unknown aetiology

**DOI:** 10.1007/s42452-022-05113-w

**Published:** 2022-07-27

**Authors:** Theophilus C. Davies

**Affiliations:** Present Address: Faculty of Natural Sciences, Mangosuthu University of Technology, 511 Mangosuthu Highway, 4031, KwaZulu Natal, South Africa

**Keywords:** Unknown aetiology, Geochemical perturbations, Immune system, Disease risk mapping

## Abstract

**Abstract:**

The term *diseases of unknown aetiology* (DUA) or *idiopathic diseases* is used to describe diseases that are of uncertain or unknown cause or origin. Among plausible geoenvironmental co-factors in causation of DUA, this article focusses on the entry of trace elements, including metals and metalloids into humans, and their involvement in humoral and cellular immune responses, representing potentially toxic agents with implications as co-factors for certain DUA. Several trace elements/metals/metalloids (micronutrients) play vital roles as co-factors for essential enzymes and antioxidant molecules, thus, conferring protection against disease. However, inborn errors of trace element/metal/metalloid metabolisms can occur to produce toxicity, such as when there are basic defects in the element transport mechanism. Ultimately, it is the amount of trace element, metal or metalloid that is taken up, its mode of accumulation in human tissues, and related geomedical attributes such as the chemical form and bioavailability that decisively determine whether the exerted effects are toxic or beneficial. Several case descriptions of DUA that are common worldwide are given to illustrate our knowledge so far of how trace element/metal/metalloid interactions in the *immune system* may engender its dysregulation and be implicated as causal co-factors of DUA.

**Article highlights:**

- The importance of a proper understanding of geochemical perturbations in human metabolisms is emphasised
- It is proferred that such an understanding would aid greatly in the decipherment of diseases of unknown aetiology (DUA)
- The thesis presented may pave the way towards better diagnosis and therapy of DUA

## Introduction

As far back as 1923 Marcus Haase noted the vast gap that exists in our knowledge of accurately identifying the cause of many diseases [1]. Despite the enormous advances made by modern medicine, there are several diseases today whose causes are still unknown (See examples in Table 1). These diseases are generally referred to as *diseases of unknown aetiology* (DUA), and as recently as 2020, an estimated seventy-six percent of unknown disease outbreaks remained undiagnosed [2]. Rappaport had earlier (2012) noted that: “Although *chronic* diseases are primarily environmental (i.e., not genetic) in origin, the particular environmental causes of these diseases are poorly understood.” [3].

**Table 1:** Examples from the global literature on diseases of unknown aetiology (DUA) having one or more possible or suspected geochemical and/or other geoenvironmental variable(s) as causal co-factor

DUA	Presentation	Incidence/Geographical Distribution/Dem-ographical Pattern	Geochemical variable (Including Trace Element Mediated Immune Response)	Geoclimatic and Seasonal Variations	Remarks
1. Acrocyanosis	*Acrocyanosis* is a peripheral vascular disorder which presents as a persistent bluish or cyanotic discolouration of the extremities, most commonly occurring in the hands	Geographical locale (latitude; urban versus rural setting) uncertain—[23]	*Chronic* As toxicity—[23–27]	Cold climate—[28, 29]; Cold environments—[30, 31]	According to Kurklinsky et al.—[23] the diagnosis of acrocyanosis remains mostly clinical; and the variegated nature of pathological mechanisms suggests that the disease is unlikely to be a single entity
2. Acute Febrile Illness	*Acute Febrile Illness* (AFI) is characterised by malaise, *myalgia* (pain in muscle or group of muscles) and a raised temperature that could be interpreted as a nonspecific manifestation of *infectious diseases* in the tropics—[32]	Sub-Saharan Africa; Tropics and Sub-Tropics	Viral respiratory tract infections—[33, 34]; Significantly lower serum Zn levels in febrile seizure group than in the afebrile group—[35]; No significant relationship observed between fabrile convulsion and the elements: Fe, Zn, Mg and Ca—[36]	Clear seasonal trend observed—[37]	Further research is warranted on trace element metabolism in relation to the development of AFI—See: [36]
3. Acute Severe Asthma	*Asthma* is characterised by chronic airway inflammation, resulting in periodic wheeze, cough and breathlessness (See: [38])	Worldwide prevalence. The Global Asthma Report of 2017 recorded a figure of approximately 334 million for the number of people in the world suffering from asthma, thus making this condition the most prevalent chronic respiratory disease—[39]	Respiratory tract infections—[40, 41]; Air pollution—[42, 43]; Decrease in antioxidant capacity in bronchial asthma as serum Se and Zn levels decrease, leading to further increase in oxidant stress and resulting enhanced inflammation and hyperreactivity in the airways—[44]; Low Se and Cu levels may have a role in bronchial asthma in Sudan, north-central Africa—[45]	Recent global rise in asthma, an early health effect of anthropogenic climate change—[46, 47]; Weather changes/cold weather—[48, 49]	The precise mechanisms by which these environmental stimuli and viruses initiate asthma or cause worsening of the disease are still unknown—[50]
4. Alzheimer’s Disease	*Alzheimer’s Disease* (AD) is the most common neurodegenerative disorder and the leading cause of dementia (i.e., the particular group of symptoms shown). It becomes worse with time (degenerative). The symptoms expressed are as a result of the damage or destruction of nerve cells (neurons) in parts of the brain involved in thinking, learning and memory (cognitive function)	By 2009, the global prevalence of dementia was estimated at 3.9% in people aged 60 + years, with the regional prevalence being 1.6% in Africa, 4.0% in China and Western Pacific regions, 4.6% in Latin America, 5.4% in Western Europe, and 6.4% in North America—[51]; Geographical variation (latitude) plays key role in dementia risk (e.g., [52])	Significantly different (ρ < 0.05) mean concentrations of Br, Cl, Ce, Hg, N, Na, P, and Rb were observed in AD bulk brain samples compared to controls—[53]; Varying trace element relationships with AD severity, with Al deposits greater in severely affected AD brain—[54, 55]; Biometal *dyshomeostasis* and toxic metal accumulations—[56–62]; Anomalous concentration levels of metals in metal-binding proteins have growth inhibition functions on neurons—[63, 64]; Trace metals and abnormal metal metabolism influence protein aggregation, synaptic signalling pathways, mitochondrial function, oxidative stress levels, and inflammation, ultimately resulting in synapse dysfunction and neuronal loss in the AD brain—De Benedictis et al. [65]; Wang et al. [66]	Air pollution, *cf.*, long-term exposure to O_3_ and PM_2.5_ above the current [2015] US EPA standards—[65] are associated with increased the risk of AD—[66]; Association between high altitude exposure, cognitive decline and dementia mortality rate—[67–71]; Associations with seasonal temperature—[72]; Global warming and *neurodegenerative disorders*—[73]	According to Thielke et al. [67] we still do not yet fully understand which environmental risk factors are associated with Alzheimer dementia; nor do we know which of these factors have links with the geological milieu In 2019 Alzheimer’s Disease International (ADI) estimated that there are over 50 million people living with dementia globally, a figure set to increase to 152 million by 2050—[74]. In both developed and developing nations, Alzheimer's disease has had tremendous impact on the affected individuals, caregivers, and society (See, e.g., [75], for some relatively recent figures for Alzheimer’s mortality in the US) Because developing countries are projected to see the largest increase in absolute numbers of older persons, their share of the worldwide aging population will increase from 59 to 71% [51]. Some authors, e.g., Qui et al. [51] believe that this dementing disorder will pose huge challenges to public health and elderly care systems in all countries across the world, because of its strong association with increasing age
5. Autism Spectrum Disorder	*Autism* *Spectrum Disorder* (ASD) refers to a diverse group of developmental conditions caused by differences in the brain, and is characterised by some degree of impaired social behaviours, speech and nonverbal communication	Worldwide prevalence. About 1 in 160 children have ASD—[76]; A geographical pattern is observed in ASD prevalence study in the US—[77]. ASD certainly prevalent in Africa, but prevalence rate is unknown, e.g., for South Africa—See: Pillay et al.-[78]; and, by 2021, very little research had been done within the school systems in South Africa	Several environmental factors mentioned in the development of ASD, include: air pollutants, fragrances, glyphosate and toxic metals, especially Al used in vaccines as adjuvant—[79]; Existence of mechanistic link between genetic mutations in *Shank proteins* and Zn deficiency in the aetiology of ASD—[80]; Significantly lower concentrations of Ca, Cu, Cr, Mn, Fe and Co in hair samples of children with ASD compared to normal children—[81]; Genetic heavy element toxicity—[56]; Zn deficiency, excess Cu levels, and low Zn/Cu ratio, common in children diagnosed with ASD—[82]; Hair concentrations of Cr, Co, I, Fe and Mg in ASD patients significantly lower than those of control subjects—[83]; Ca deficiency and toxic metal (As and Pb) overload—[84]; Children with ASD present a reduced ability of eliminating toxic metals, leading to these metals accumulating in the body—[85]; The levels of Hg, Li, Pb and Se in the hair of autistic children were higher than those of healthy children, while the levels of Zn in the blood were lower—[86]; Children exposed to O_3_, CO, NO_2_, and SO_2_ in polluted air during the preceding 1 year to 4 years may be amenable to increased risk of ASD diagnosis—Jung et al., [87] 2013	Season of birth as a risk factor for ASD—[88]	-
6. Chronic Fatigue Syndrome*/Myalgic Encephalomyelitis*	*Chronic Fatigue Syndrome*/*Myalgic Encephalomyelitis* (CFS/ME) is a disabling, debilitating and complex disease characterised by profound fatigue, sleep abnormalities, pain and other symptoms that are worsened by exertion	Worldwide prevalence—[6, 89], with 17 to 24 million people having the disease—[90]	Metal hypersensitivity—[91]; Some nutrient deficiencies (vitamin C, vitamin B complex, Na, Mg, Zn, folic acid, L-carnitine, L-tryptophan, essential fatty acids, and coenzyme Q10) appear to be important in the severity and exacerbation of CFS—[92]; Ca associated with some of the neurological findings described in ME/CFS—[93]; Insufficient Ca inflow into cells that perform intracellular functions—[94]	-	According to the US CDC—[95], researchers have still not yet been able to find the cause(s) of ME/CFS, and there are as yet no specific laboratory tests to diagnose ME/CFS directly. It cannot be fully explained by an underlying medical condition More epidemiologic studies are needed on the prevalence and sociodemographic characteristics of CFS in developing countries—[96] Sierpina and Carter—[97] suggested that 200 mcg of chromium picolinate (taken with meals) may have the potential to reduce any reactive hypoglycaemia that may aggravate the symptoms of CFS
7. Chronic Kidney Disease of Unknown Aetiology	*Chronic Kidney Disease of unknown aetiology* (CKDu) has, as its predominant feature, tubular atrophy and interstitial fibrosis (thickening and scarring of the tiny air sacs and interstitial tissues in the lungs)	Reported in many parts of the world, especially among rural farming communities. High incidence in low- and middle-income countries over last two decades—[98, 99]; “In 2017, the global prevalence of Chronic Kidney Disease (CKD) was 9·1% (95% uncertainty interval [UI] 8·5 to 9·8), which is roughly 700 million cases”—[100]	Synergistic reaction between Cd and diabetic-related hyperglycaemia—[101]; Consumption of (polluted) well water suggested; need for investigating role of Cd—[102]; Too high Ca intake?—[103]; “Geographical mapping showed that villages with a high prevalence of CKDu are often related to irrigation water sources and/or located below the level of the water table” [104]; Toxins/heavy elements -[105]; Groundwater geochemistry (high levels of F^−^, Cd, As)—[106]; Exposure to low levels of Cd—[107]; High ionicity of drinking water due to fertiliser runoff -[108, 109]; Toxic metal exposure; water pollution—[110]; Synergistic reaction between fluoride and water hardness—[111–113]; Chemical species such as Ca, phosphate, oxalate, and F^−^ form intra-renal nanomineral particles initiating the CKD of multifactorial origin (CKDmfo)—[114]; Total dissolved solids and As in drinking water have a positive correlation with CKDu—[115]	Altitude—[116]; Heat stress nephropathy due to global warming—[117–119]; Climatic patterns—[114]; A quintessential climate-sensitive disease—[120] Salas et al., 2019	In 2017 Gifford et al. [121] noted that of the several epidemics of CKDu that have occurred worldwide, some, like Itai-Itai disease in Japan and Balkans nephropathy have been explained, whereas the aetiology of others remains unknown There is the absence of common risk factors or underlying conditions that lead to CKD, such as diabetes, immune-mediated glomerulonephritis, or structural renal disease—Caplin et al.—[122]
8. Endomyocardial fibrosis	*Endomyocardial Fibrosis* (EMF) is a form of restrictive cardiomyopathy of unknown aetiology, characterised by endocardial fibrosis of the apices and inflow tracts of the right ventricle, left ventricle or both—[123]	Most prevalent in the tropical regions of Africa, Asia, and South America, and mainly affecting young adults of lower socioeconomic status in those regions	Significantly elevated Ce levels (ρ < 0.05) in serum of EMF patients compared to controls—[124]; Ce contamination in soil and water—[125]; High levels of Ce; Deficiency of Mg promotes the absorption of Ce and enhances its toxicity forming the basis for the initial injury of the heart—[126–128])	-	In 2014, Mocumbi considered that in the field pf cardiovascular medicine, EMF is perhaps the most neglected disease; and despite its high prevalence rate in Africa, Asia and South America, few human and material resources are made available in these regions for research on its mechanism—[129] Today (2021), the exact aetiology and *pathogenesis* of EMF still remains unknown (See e.g., [123, 125, 129])
9. Fibromyalgia	*Fibromyalgia is* a rheumatic condition characterised by chronic pain, fatigue, and tenderness of muscles, tendons, and joints; often accompanied by fatigue, sleep, memory and mood issues	*Fibromyalgia has a worldwide* prevalence ranging from 0.2% to 6.6% in the general population; in women between 2.4 and 6.8%; in urban areas between 0.7 and 11.4% in rural areas [130]	An imbalance of the trace element status in human tissues and body fluids—[131, 132]; Metal-induced oxidative stress contributes to the severity of FMS – [133], [134]); Elevated blood Pb and Cd levels in FMS patients, compared with control group; Serum Ca and Mg levels significantly reduced (ρ < 0.05) in FMS patients compared to control group—A [135]; Women with FM have lower dietary intake of Ca, Mg, Fe and Mn in comparison with women who did not have the condition—[136, 137]	Fors and Sexton’s 2002 study—[138] did not reveal any statistically significant relationship between fibromyalgic pain and the weather, although it is possible that certain patients with less chronic fibromyalgia might be weather sensitive	The aetiology and *pathogenesis* of fibromyalgia till today (2021), remain a mystery. Several proposed co-factors such as dysfunction of the central and autonomic nervous systems, neurotransmitters, hormones, immune system, external stressors, psychiatric aspects, and others are still being researched (See e.g., [139]. The recent literature clearly shows that the role of the *metallome* in FMS aetiology deserves far more attention
10. Geographic Tongue	*Geographic tongue* (also known as *benign migratory glossitis*) is an inflammatory disorder that usually appears in a map-like (geographic) pattern on the dorsum and margins of the tongue. Typically, affected *tongues* have a bald, red area of varying sizes that is surrounded, at least in part, by an irregular white border	A common condition, affecting 2–3% of the adult general population, *worldwide*—[140, 141]	Fe and Zn deficiency; Vitamin B12—[142–145]; Low levels of salivary Zn in affected individuals compared to control groups -[146]	-	-
11. Ill-thrift or ‘Unthriftiness’ as it is called in South Africa	*Ill-thrift* or *Unthriftiness* is an ill-defined condition of young sheep. Affected animals show mild to severe depression of growth rate and anaemia	In South Africa, the disease occurs in the coastal areas of the Eastern Cape Province. The condition has been reported from a number of other countries, including Australia, New Zealand, France and Norway—[147]	Cu, Co, Se and I, being essential components of the diet of beef cattle for maintaining their health and productivity, their deficiency in these elements can cause ill-thrift and infertility, among other causes—[148]; A state of sub-optimal growth (ill-thrift) in buffalo-calves was largely attributed to trace element deficiency, in particular Cu, Co, Fe, Se and Zn deficiency that may cause reduction in the total antioxidant capacity, with a lower ability to reduce oxidative compounds—[149–151]	Mainly reported from coastal areas of high rainfall—Examples are found in Australia—[151] and South Africa [147]	-
12. Kawasaki Disease	*Kawasaki Disease (KD)* is an acute, self-limited *vasculitis* **(inflamed blood vessels)** of infants and children, with unknown aetiology Signs of KD include prolonged fever associated with rash, red eyes, mouth, lips and tongue, and swollen hands and feet with peeling skin. The disease causes damage to the coronary arteries in a quarter of untreated children and may lead to serious heart problems in early adulthood	KD occurs worldwide; most prominently in Japan, Korea, and Taiwan, reflecting increased genetic susceptibility among Asian population—[152] The epidemiology of KD in Africa is very ill-defined, which inevitably leads to misdiagnosis and the reporting of very few cases. This gives the impression that the condition is rare in Africa—[153–155]. The presentation of KD is similar to that of measles (which is very prevalent in Africa), so the exact prevalence (of KD in Africa) is difficult to ascertain—See e.g., [153, 156]	Environmental exposure to Hg—[157–160]; Airborne pathogens or toxins—[161, 162]	Seasonality of KD, with winter peaks and winter-spring predominance in Japan and the US, respectively, and in many other temperate areas—[152]. Decades of research have been unable to unearth the cause of the disease, but its distinct seasonality can hardly be in doubt—[163] -[167]	(i) Hara et al.—[20] noted in 2016, that the contribution of environmental factors is greater in the development of KD than genetic factors among individuals with the same ethnicity (ii) The temporal association between the COVID-19 pandemic and the results of RT-PCR and antibody testing suggests a causal link between Kawasaki disease and COVID-19—[168] (iii) As Rowley and Schulman remarked in 2018—[152] “The occurrence of epidemics and geographic wave-like spread of KD during epidemics supports a presently unknown single agent or closely related group of agents as the etiology.” [*Sic.*]
13. *Lupus erythematosus*	*Systemic Lupus erythematosus* (SLE) is a chronic inflammatory autoimmune disease of multifactorial origin—[169]	Worldwide. Highest prevalence rate in North America—[170]. Once thought to be of low prevalence rate in Sub-Saharan Africa (due to under-reporting?) SLE prevalence rate is now found to be 1.7%—[171], as a result of availability of improved diagnostic capacity	Low serum levels of albumin, Zn, Se and Zn/Cu ratio; negative correlation between serum Cu levels and lupus disease activity—[172]; SLE patients have different profiles of trace elements and toxic metals compared to healthy controls—[169]	“Active SLE has the characteristics of seasonal distribution and is associated with temperature. The mechanism remains to be further studied”—[173]	The aetiology of SLE is complex, and incompletely understood (See: [174]). “More epidemiological studies in Africa are warranted.”—[170]
14. Multiple Sclerosis	*Multiple Sclerosis* (MS) is a demyelinating disease (a nervous system disease in which the insulating covers of nerve cells in the brain and spinal cord are damaged). This damage disrupts the ability of parts of the nervous system to transmit signals, resulting in a range of signs and symptoms, including physical, mental, and sometimes psychiatric problems—[175–177]. Kister et al. [178] list 11 specific symptom domains commonly affected in multiple sclerosis: mobility, hand function, vision, fatigue, cognition, bowel/bladder function, sensory, spasticity, pain, depression, and tremor/co-ordination—[178]	Distribution is worldwide. Distinct geographical pattern of prevalence with high prevalence rates between 45 and 65 degrees north—[179]. The age-standardised MS prevalence estimate per 100,000 population for eastern Sub-Saharan Africa is put at 3.3 by WGBD (2019)—[180]; but such an estimate would always be influenced by misdiagnosis and under-reporting (See, e.g., [181])	Metabolic imbalance of trace elements/metals—[182–188] the therapeutic potential of antioxidant [*reactive oxygen species* (ROS)] protection in the pathogenesis of MS—[189, 190] Effect on the immune system of Al toxicity and Cu, Zn, and Se toxicity and deficiency, followed by neuron inflammation and degeneration—[191]	People living in higher geographical *latitudes* may receive lower levels of sunlight, and therefore have lower vitamin D levels which probably explains the reason for a higher incidence of MS in countries with higher *latitudes—*[192]. “There is a striking latitudinal gra-dient in multiple sclerosis (MS) prevalence …”—[193, 194] See also: [195–197]	-
15. Nodding Disease (ND)/Nodding syndrome (NS)	*Nodding Disease* is characterised by an occasional nodding of the head, as in epilepsy, with seizures, stunted growth, and with mental retardation sometimes occurring	This is an emerging disease occurring in *clusters* in South Sudan, southern Tanzania, northern Uganda and possibly also other countries of Sub-Saharan Africa. The exact prevalence and geographic distribution of the disease in the affected countries is still unknown—[198]	Deficiency of vitamin B6 (pyridoxine) and other micronutrients such as vitamin A, Se, and Zn—[199, 200]. Nutritional toxicity—[204]	Climate change—[201]; Cold weather—[202, 203]. Living in the vicinity of fast-flowing streams, the breeding habitat of the black fly—[201]	As at 2020, several aspects of NS remained unclear, a feature that Olum—[205] considered unsurprising, given the existence of so many acquired neurological diseases whose aetiology is not well understood
16. Noma	*Noma* (*cancrum oris* or *gangrenous stomatitis*), is a severe and progressive gangrenous infection (body tissues die as a result of infection or inadequate blood supply) that affects the mouth and face	Mainly observed in tropical countries, particularly those in Sub-Saharan Africa. True global incidence unknown; but estimated incidence of 30,000—40,000 has been suggested by Srour et al.- [206]	Deficiencies of trace elements and amino acids influencing the efficacy of the immune system: Fe, Zn, cysteine, methionine, serine, and glycine—[207] Deficiency of micronutrients in the diet—[206–210]	-	Patients generally live in extremely poor conditions, frequently located in remote rural areas
17. Parkinson’s Disease	*Parkinson’s Disease* (PD) is a progressive heterogeneous, multisystem and neurodegenerative nervous system disorder that affects movement. The cardinal features of Parkinson's disease are (i) tremor, mainly at rest; (ii) muscular rigidity, which leads to difficulties in walking, writing, speaking and masking of facial expression; (iii) *bradykinesia*, a slowness in initiating and executing movements; and (iv) stooped posture and instability—[211] Parkinson's disease occurs when nerve cells, or dopamine-rich neurons in an area of the brain that controls movement called the *substantia nigra*. become impaired and/or die. But the complete series of steps leading to this cell death is still vague, and the underlying causes remain one of medicine’s greatest mysteries	Worldwide occurrence. According to the 2016 Dorsey and GBD Collaborators Study—[212] published in 2018, 6·1 million (95% uncertainty interval [UI] 5·0 -7·3) individuals had Parkinson's disease globally, compared with 2·5 million (2·0—3·0) in 1990; Geographical variation (*cf*., latitude) plays key role in dementia risk (e.g., Russ et al.—[52]	High concentrations of Al and low levels of Mg observed in the pathogenesis of CNS (central nervous system) degeneration and PD—[213]; Association with metal and trace element concentration in urine, serum, whole blood and cerebrospinal fluid—[214–218]; Very high or very low levels of Se—[219] Combination of Mo deficiency and purine ingestion—[220] Significant association between the PD mortality rates and soil concentrations of Se, Sr, and Mg—[221–223]; Elevated trace metals (namely, Cu, Zn, Mg and Fe) found, compared to controls (ρ < 0.001) in Nigerian Parkinson’s Disease patients—[224]	Existence of seasonality (related to temperature) in Parkinson's disease symptoms—[225]; Improvement of PD symptoms at high altitude—[226, 227]; Several risk factors in development of PD at high altitude—[228]	Regional maps depicting correlations between the distribution (clusters) of PD and soil geochemistry which would be helpful in this aetiological debate; but are very rare in the published literature (See an example in: Sun—[221] for PD distribution in the United States) To date, according to Ullah et al.—[229], the fundamental molecular mechanisms of PD aetiology and pathogenesis remain unclear, with a number of epidemiological studies implicating metal toxicity in the diseases’ pathogenesis via several potential mechanisms. In many instances, the metal equilibrium is thought to be disturbed, leading to deleterious effect on the entire body including the brain
18. Sarcoidosis	*Sarcoidosis* is a multisystem, granulomatous, inflammatory disease that affects one or more organs, but most commonly affects the lungs and lymph glands. The inflammation may change the normal structure and possibly the function of the affected organ(s)	*Sarcoidosis* is observed throughout the *world* and affects all races and ages, with an average incidence of 16.5 per 100,000 in men and 19 per 100,000 in women [230]. Race and geographical location are considered as factors in the incidence and prevalence of sarcoidosis, which has consistently been observed to be highest in Nordic countries and in African Americans—[231]. Sarcoidosis in not a rare condition in Africa—[232, 233], the apparent infrequency of reporting being probably a result of misdiagnosis (as, e.g., tuberculosis)—[234, 235]. Further research is therefore necessary in Africa to unravel the various clinical aspects of this mysterious and complex disease	Exposure to toxic metals and rare earth elements (REEs) in the environment—[236–238]; Metal dusts—[239]; Crystalline silica—[231]; Industrial exposure to Be—[240] Since low dose metal particles can induce *monocyte*/*macrophage* survival (See e.g., Lacey et al.—[241]), as recently as 2021, Lepzien et al.—[242] went on to show that *monocytes* could be a vital marker in understanding the inflammatory process of sarcoidosis	Geographic clustering of disease in many parts of the world has long been noted, [e.g., in the US (Sartwell and Edwards—[243]); this (clustering) has ignited further speculations concerning weather, soil, and foliage in the pathogenesis of sarcoidosis. More recently (2019), Ramos-Casals et al.—[244] asserted that local weather is a key environmental factor influencing the incidence of sarcoidosis in a specific geographical area, with the peak of diagnosed cases following a specific seasonal distribution pattern	The causes of sarcoidosis are still unknown and epidemiological data are often discordant—[245, 246] Although the aetiology of this condition remains uncertain, Ganeshan et al. [247] consider that the role of environmental and genetic factors may be considerable in any proposed causative mechanism According to Ahmadzai et al.—[248], in sarcoidosis, conventional sampling techniques or cultures of non-caseating granulomas cannot detect tissue micro-organisms; although as Newman earlier (1998)—[236] proposed, clinicians should use a systematic approach to investigating the occupational and environmental history and immunologic responses of patients with sarcoidosis, for discriminating metal-induced granulomatosis from sarcoidosis
19. Spastic Paraparesis	*Hereditary Spastic Paraparesis* (HSP) or the *Strümpell-Lorrain Syndrome* refers to a heterogeneous group of disorders in which the main clinical feature is progressive lower limb spasticity or gait disorder HSP is also known as *familial spastic paraplegia*—[249]; **however, “***paraparesis*” indicates weakness in both legs, and is of lesser severity than “*paraplegia*”	The prevalence of all hereditary spastic paraplegias combined is estimated to be 2 to 6 in 100,000 people—[250]	Nutritional disorders, including Cu deficiency, vitamins B_12_ and E deficiencies—[251–253]	High intake of HCN^−^ engendered by drought conditions—[254]	See Taibo et al.- [255] for report of a real-life outbreak of spastic paraparesis investigation undertaken in northern Mozambique in 1981
*20. Sudden Infant Death Syndrome*	*Sudden Infant Death Syndrome (SIDS)* is the sudden and unexplained death of a baby younger than 1 year old, after thorough case investigation—[256]	Has a global distribution—[257]; A significant cause of mortality in Africa (See: Ogbu—[258]; Ndu—[259]; and Dempers et al.—[260]; and many of the risk factors of SIDS exist. The syndrome may well be widespread in Africa (See, e.g., Ogbu—[258]; Ndu—[259]; but scarce attention is given to research on it	Biodeterioration of cot mattresses from extracellular enzymes of *Streptococcus brevicaulis* fungi, microorganisms that are capable of converting preservative plasticisers and fire retardants to arsine and phosphines—[27]; Genetic heavy element toxicity—[56]; Elevated Pb burden (blood samples) in SID babies compared with control babies—[261]; Increased tissue Pb levels in SIDS infants—[262]; K levels significantly lower in less than 6-month-old SIDS infants than in non‐SIDS infants—[263]; Soft water with low Mg and Ca and with high concentration of Na, linked to higher SIDS rates—[264]; Recharge of groundwater which increases its nitrate content—[265]	Cold wet weather—[266] Overheating or disordered thermoregulation—[267] Exposure to increased ambient *temperature* associated with an increased risk of *SIDS—*[268, 269]	“Despite decades of investigation and millions of dollars spent, the cause of sudden infant death syndrome (SIDS) eludes researchers. It is timely therefore to reconsider the reasons for this failure and to explore how research might go forward with better prospects.” [*Sic.*-270] In deciphering the causes of SIDS, we must remember that a *baby’s immune system* is immature at birth, making newborns particularly at risk of illness (See e.g., Goenka and Kollmann—[271]; Simon et al.—[272]. It is therefore critically important that their potential contact with geonvironmental factors of disease, especially the *metallome*, be given particular attention by researchers and caregivers

Source: Adapted and expanded from [273] (With permission)

A recent World Health Organisation (WHO) study of worldwide cancer mortality identified ten diverse environmental *risk* factors, including some with links to the geological environment, such as air pollution and ionising radiation exposure [4]. But on the whole, it appears that the influence of geoenvironmental factors in disease causation, in particular, the effects of involvement of trace elements/metals/metalloids in human metabolisms that produce disease, has been somewhat underestimated.

In this paper, it is argued that greater consideration should be given to the contribution of the geoenvironmental co-factor in a multi-factor explanation and diagnosis of DUA; in particular, the entry of trace elements including metals and metalloids into biological systems, and their involvement in humoral and cellular immune responses. Possible ways in which trace elements/metals/metalloids can contribute towards shaping developmental metabolic frameworks and pathways in DUA progression are described. The expectation is that a firm understanding of the role of geoenvironmental co-factors, more especially, trace element/metal/metalloid perturbations that produce errors or disturbances in metabolic processes will help greatly in unravelling the aetiology of DUA when this knowledge is applied in a circumspective way.

Rationalisation of such knowledge falls within the compass of the Medical Geologist who, according to Bundschuh et al. [5], can provide credible explanations regarding the mode of occurrence, mobility, bioavailability and bio-accessibility characteristics, as well as exposure and transfer mechanisms of *geochemicals* to the food-chain and humans; and the nature of related ecotoxicological and health effects that are produced.

To these parameters, can be added the chemical form of the element, a parameter that, in turn, greatly influences mobility, bioavailability and the mechanism that either transports the element to the centres (of the body) where it is needed for vital reactions, or be involved in interactions that result in disease (*cf.*, DUA). An approach that integrates all possible or suspected co-factors is contingent upon the realisation that most DUA and other enigmatic diseases have multifactorial causes, engendering a complex networking between genetic factors (polygenic), immunological mediators (trace elements/metals/metalloids) and various other (geo)environmental factors, none of which factors would cause the disease on its own.

This approach is further buttressed by Panelli’s (2017) observation that we need to look more closely at multisystem diseases of unknown cause and *seek new ways to diagnose and discriminate diseases whose aetiologies are still unclear, but are* affecting large populations of patients worldwide [6].

### Content

The paper is divided into eight major sections. The first of these, “Introduction”, brings to awareness the myriad of globally occurring diseases whose causes to date, are still imprecisely known. A demonstration is made of how *metallome* disturbances negatively affect the immune system and how a proper understanding of geochemically-related perturbations in human bodies might provide useful clues for improving diagnosis and therapy of DUA. This Section also incorporates the “Methodology”, which is based on an iterative approach in a comprehensive search and review of pertinent documents on ‘unknown aetiologies’ from a number of key databases.

In Sect. 2, brief explanations are given on how best to obviate classifying an observed association between a risk factor and disease (*cf.,* DUA) as due to chance (random error), bias (systematic error) or confounding. The role of various geoenvironmentally-related variables as co-factors in disease causation is briefly reviewed in this Section.

In Sect. 3, brief discussions are given of how geochemical variables can have profound effects on biological systems, using the examples of ‘speciation’, ‘variations in natural isotopic ratios in tissues’, and ‘bioavailability’.

Section 4: “Geochemical variables and the immune system” presents a comprehensive review of several aspects of immune system function and autoimmune diseases (AuDs), since a number of DUAs fall under this category.

Section 5: “Criticality of the optimal range of intake and the occurrence of nutrient toxicities” emphasises the importance of optimum level of nutrient-uptake, and recommends nutritional measures that could be taken to maintain the correct level.

Section 6: “Disease risk mapping and DUA cluster detection” discusses the importance of these maps, and how they can be applied to the identification and analysis of clusters of DUA; hence providing clues on their origin.

Section 7: “Conclusions”, gives the main conclusions drawn from the study, exposes gaps in knowledge and recommends some urgent areas of research into DUA.

Section 8: “Glossary of Terms” presents definitions and explanations of technical terms, abbreviations and phrases that are unfamiliar to non-medical scientists and others from allied fields of the multidisciplinary science of ‘Medical Geology’.

### Methodology

An iterative approach was adopted in a comprehensive internet search through October 10, 2021, combining the results from multiple search engines—Google scholar, PubMed, ScienceDirect and SpringerLink—to achieve an improvement in the analysis of each dataset. Initial searches used broad terms: ‘geo-environmental factors’, ‘unknown aetiology’ and ‘disease X’. Inclusion criteria were accounts of studies carried out in humans and animals and reported observational designs. The documents returned from these searches were used to identify narrower search terms, such as ‘risk factors’, ‘nutritional and toxic elements/metabolic imbalances’, ‘immune system’. Over seven hundred documents were retrieved (including some duplicates), out of which, conclusions from about four hundred and sixty were studied in detail. These included peer reviewed journal articles and conference proceedings, authentic book chapters, published and unpublished theses and reports, and selected web references.

## Causality

In the field of medicine*, cause*, also sometimes referred to as *aetiology* is the reason or origination of a disease, or of a *pathology* (essential nature of disease) [7]. The word ‘aetiology’ stems from the Greek *αἰτιολογία, aitiologia*, “giving a reason for” (*αἰτία*, *aitia*, “cause”; and—*λογία*,—*logia*) [8].

Attempts at unravelling the aetiologies of human diseases go back as far as to antiquity. Hippocrates, a Greek physician of the fourth and fifth centuries BCE, is believed to be the first to adopt the concept that disease is not a visitation of the gods but rather, results from earthly influences [9]. Medieval European doctors were generally of the view that disease was related to the *air* and adopted a *miasmatic approach* to disease aetiology [10]. Scientists from the field of medicine and from allied sciences have since continually searched for the causes of disease and, indeed, have discovered the causes of many. Where no definite aetiological characterisation can be made, the disorder is said to be *idiopathic.*

Traditional accounts have linked the causes of disease to the *evil eye*, a phenomenon elucidated by Abu-Rabia in 2005 [11], in describing the rituals of diagnosis, treatment and prevention among the Bedouin tribes of the Negev in the Middle East.

In medicine, debates on the history of aetiological discovery always make reference to Robert Koch’s affirmation in 1882, that the tubercle bacillus (*Mycobacterium tuberculosis* complex) causes the disease tuberculosis, *Bacillus anthracis* causes anthrax, and *Vibrio cholerae* causes cholera [12].

This ideation and affirmation is encapsulated in Koch’s notions. In epidemiological research on infectious diseases, proof of causation is limited to individual cases where evidence of aetiology can be demonstrated experimentally. In order to infer causation, we require several lines of evidence, taken together.

### Chain of causation and correlation

**Fig. 1:**
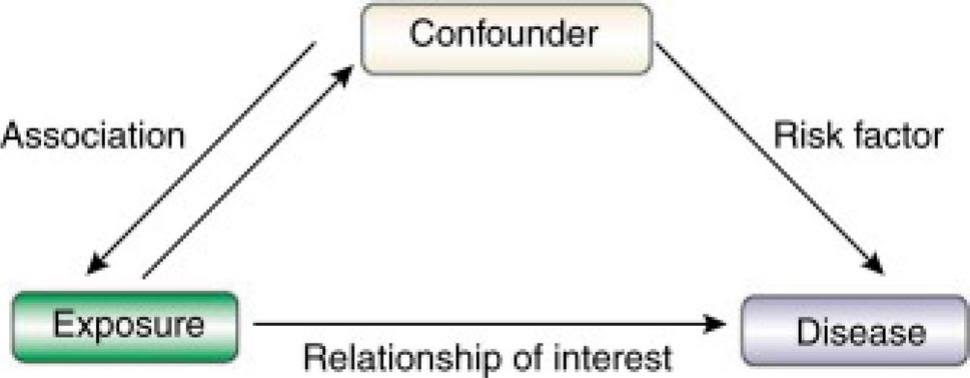
The structure of confounding. Source: Jager et al. [13]

We need to distinguish between *causation* and *association* or *statistical correlation*. Events may occur simultaneously simply due to *chance*, *bias* or *confounding* (See: “Glossary of Terms”, this article, for definitions), instead of one event being precipitated by the other. It is also necessary to decipher which event is the cause. *Confounding* is said to occur when exposure to a probable disease causative agent or cofactor and an outcome have an apparent but false correlation (Fig. 1). It is important to control for the confounder, otherwise, there would seem to be a link between the exposure and the outcome, when in fact both are due to the confounding effect and bear no relationship at all (or no strong relationship). Careful sampling and analyses should be the *sine qua non*, rather than complex statistical analysis to establish causation. Evidence garnered from experimental studies involving interventions (providing or removing the supposed cause) provides the most convincing evidence of aetiology.

It is also necessary to state that there are times when several symptoms appear together, sometimes more than what could be expected; though it is known that one cannot cause the other. These situations are referred to as *syndromes* (See “Glossary of Terms”, this article). The assumption is that an underlying condition exists that explains all the symptoms. Quite often, however, a single cause for a disease cannot be found, but rather, we find a *chain of causation* from an initial trigger to the development of the clinical disease. An aetiological agent of disease may require an independent co-factor and be subject to a *promoter* (See “Glossary of Terms”, this article) to cause disease.

### The geo-environment as an agent of disease

The causal co-factors of disease occurrence and progression are legion, and include genetics, microbes/fungi, environmental factors such as exposure to geogenic contaminants (geochemicals, xenobiotics), geographical patterns, seasonality, climate change, geopathic stress, heat waves and heat stress and spacio-temporal associations. In any discussion on the determinants of heath, the effect of socio-economic factors such as education, income and wealth, should never be overlooked, for they shape our health in important ways, not least, in providing clues on likely pathways and mechanisms that may explain their effects.

In 2000, Kroll-Smith et al. noted that: “Struggles over environmentally induced diseases are struggles over the very nature of what exists and how we know the nature of the phenomenon” [14]. Suggestions that the geoenvironmental milieu (geographical and climatic patterns, seasonal variations, geological and geochemical variables) can have a significant influence on the occurrence and development of disease, has for long captivated scholarly attention across a number of disciplinary and policy domains. Mehri, for instance, discusses how geoenvironmental conditions work in concert with infectious agents that activate *innate* and *adaptive* immune system (See “Glossary of Terms”, this article) and provoke DUA in genetically susceptible patients [15].

Geochemicals such as metals, metalloids, and radionuclides, as well as transuraniums, referred to as *geogenic contaminants* (GCs) by Bundschuh et al. [5], occur naturally in geogenic sources (e.g., minerals, rocks, ground- and surface waters and volcanic emanations). Their accelerated release globally has been attributed to rapid population rise and economic growth, and the associated increase in demand for water, energy, food, and mineral resources. The release of GCs occurring in near surface environments can be triggered into the soil, water, air and biota compartments, and subsequently enter the food chain, with often deleterious health consequences.

Writing on one of the more well-known DUAs [*chronic kidney disease* (CKD): Table 1), Hara et al. [16] remarked on the significance of the contribution of environmental factors compared to genetic factors in the development of CKD among individuals with the same ethnicity. In 2017, Senanayake and King, reviewing recent research done on emerging health-environment relationships, categorised the studies done into three themes, viz: complexity, uncertainty, and bodies [17]. Although there have been robust contributions to these thematic areas from geography and the social sciences, Senanayake and King [17] construe that integrating them (contributions) into an analytical framework can extend geographical perspectives on scale, knowledge production, and human–environment relations, while also incorporating valuable insights from cognate fields.

The cardinal thesis here is that proper consideration of geoenvironmental co-factors -more especially the geochemical-, can significantly contribute to resolution of causation of DUA, probably to an extent greater than what has hitherto been conceived (Table 1). Some examples of probable geoenvironmental and related co-factors to be considered are:

(i) The immune-modulatory effect of geochemical variables (e.g., chemical form, the mechanism of element transport and bioactivity) that underline nutritional and potentially toxic element (PTE) perturbations in metabolic processes (See, e.g., Lukác and Massányi [18]).

(ii) The production of reactive oxygen species (ROS) and DNA damages wrought by metabolic imbalance of trace elements/metals/metalloids (disruption of metal ion homeostasis) (See, e.g., Juan et al. [19]).

(iii) Water, soil and air pollution emanating from diverse sources that include volcanic emissions, mining, naturally contaminated groundwater, radon emanations into buildings, agriculture and industry. A substantial part of the pollution load from these sources often comprises the PTEs (e.g., arsenic, fluorine, mercury and lead) having a propensity to enter the food chain (through consumption of food crops and drinking water, as well as through other intake pathways such as inhalation and direct contact) [*cf*., (i) above]. Initially undetected release of a chemical from the Earth’s sub-surface into the groundwater system can occur, such as when CO_2_ gas was released in the Lake Nyos (Cameroon) disaster of the 1980’s (See, e.g., *Rouwet *et al*. *[20]*;* Boehrer et al. [21]*).*

(iv) Geogenic dust particles from mining, ore processing and vehicular transportation on untarred roads.

(v) Over-exposure to ionising radiation and radionuclides in the water, soil and air environments during mining, ore processing and tailings handling of uranium, gold and other radiogenic ores (See, e.g., US EPA [22]).

(vi) Geographical patterns (e.g., locality, altitude) and seasonal variations.

(vii) Climate change and *geoclimatic* effects.

(viii) Factors of *geopathic stress* and *heat stress.*

### Role of genetics

A *gene* is the basic physical unit of heredity. Genes are made up of *DNA* (deoxytribonucleic acid) and act as instructions to synthesise molecules called *proteins*. Many proteins are actually *enzymes*, and are responsible for carrying out all cellular functions. Salzberg estimated the number of genes in the *human genome* (genetic complement) to be 20,000 to 25,000 [274]. Genes are passed on from parents to offspring, and contain the information needed to specify traits.

There are a number of human diseases that result from mutations in the genetic complement residing in the DNA of chromosomes. Although mutations occurring in the DNA of somatic (body) cells cannot be inherited, they can cause congenital malformations (existing at birth) and cancers. Mutations that occur in germ cells, viz., the gametes, ova and sperm, are passed on to offspring causing inherited diseases.

Studies on how environmental exposures modify the expression of genes without directly changing the genetic code stored in DNA were appraised by Rappaport in 2016 [275], and more recently by Perera et al. in 2019 [276]. Such studies belong to the field of *environmental epigenetics*, a field that is currently being actively researched by the United States National Institute of Environmental Health Sciences (US NIEHS) [277].

**Fig. 2:**
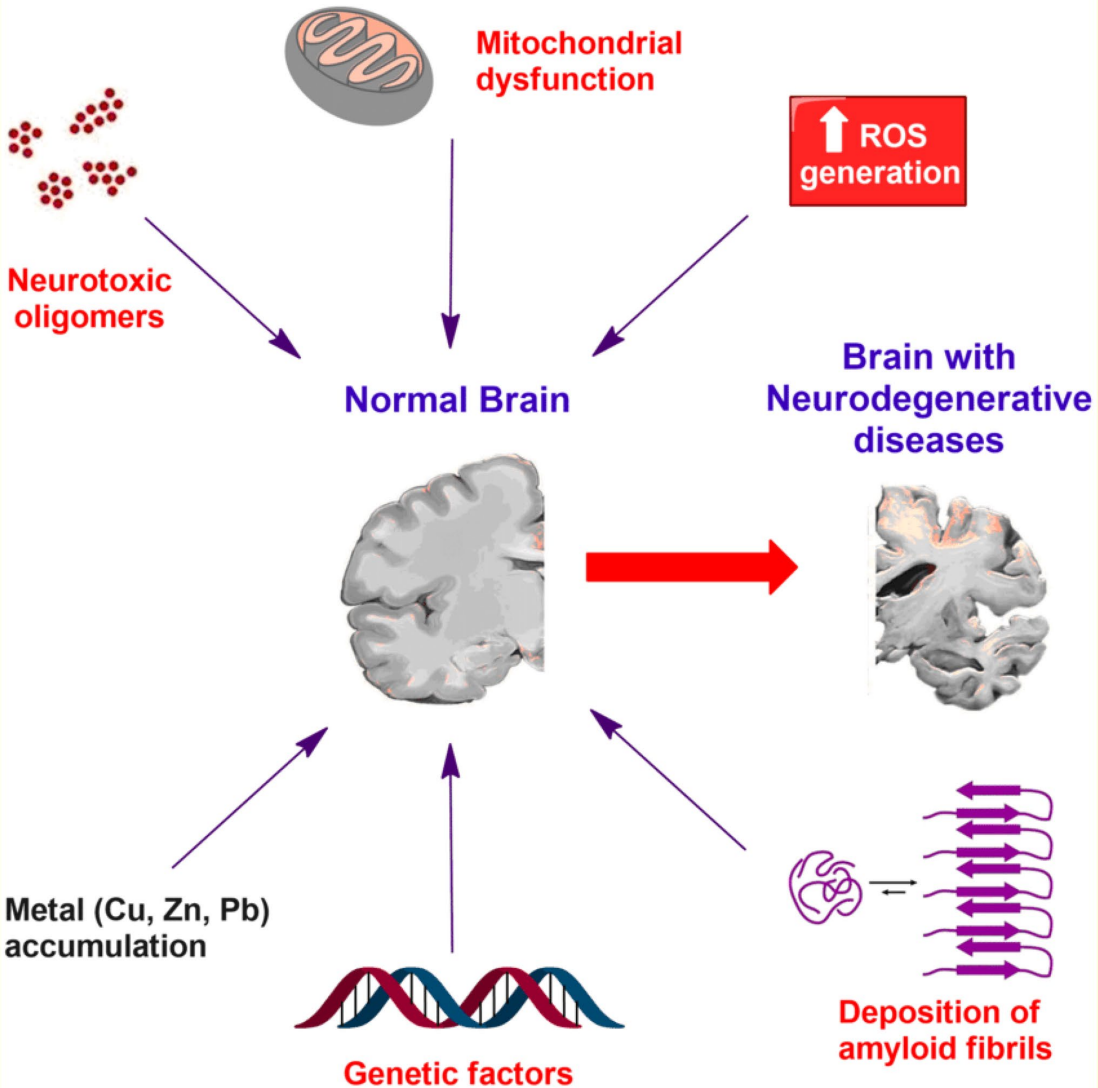
The complex and multifactorial nature of neurodegenerative DUA, and the position of the metallome (exemplified by Cu, Zn and Pb) in their development. Credit: Moustafa Gabr; Source: Ibrahim and Gabr [283]. Reproduced under the Creative Commons Attribution-NonCommercial-ShareAlike 4.0 License

Although the principle biological function of DNA is the storage of genetic information, its unique chemical structure renders this molecule amenable to metal binding via both the phosphate backbone and nucleobases or both (Kanellis and Dos Ramedios [278]. Binding of metals to the bases usually disrupts base pair hydrogen bonding and destabilises the double helix (Anastassopoulou [279]). Research on the role of DNA-bound metal ions in the incidence of certain DUA such as the neurogenerative diseases (e.g., AD, PD and MS) has been going on with increased intensity in the last two decades (See, e.g., Anastassopoulou [279]; Dales and Desplat-Jégo [280]; Morris, Jr. [281]; Hasani Nourian et al. [282]), *but exact pathways and mechanisms by which metal toxicity is induced are still not fully understood*. (Ibrahim and Gabr [283]) and a number of other authors consider it likely that each metal could be toxic through specific pathways and mechanisms (See Fig. 2).

**Fig. 3:**
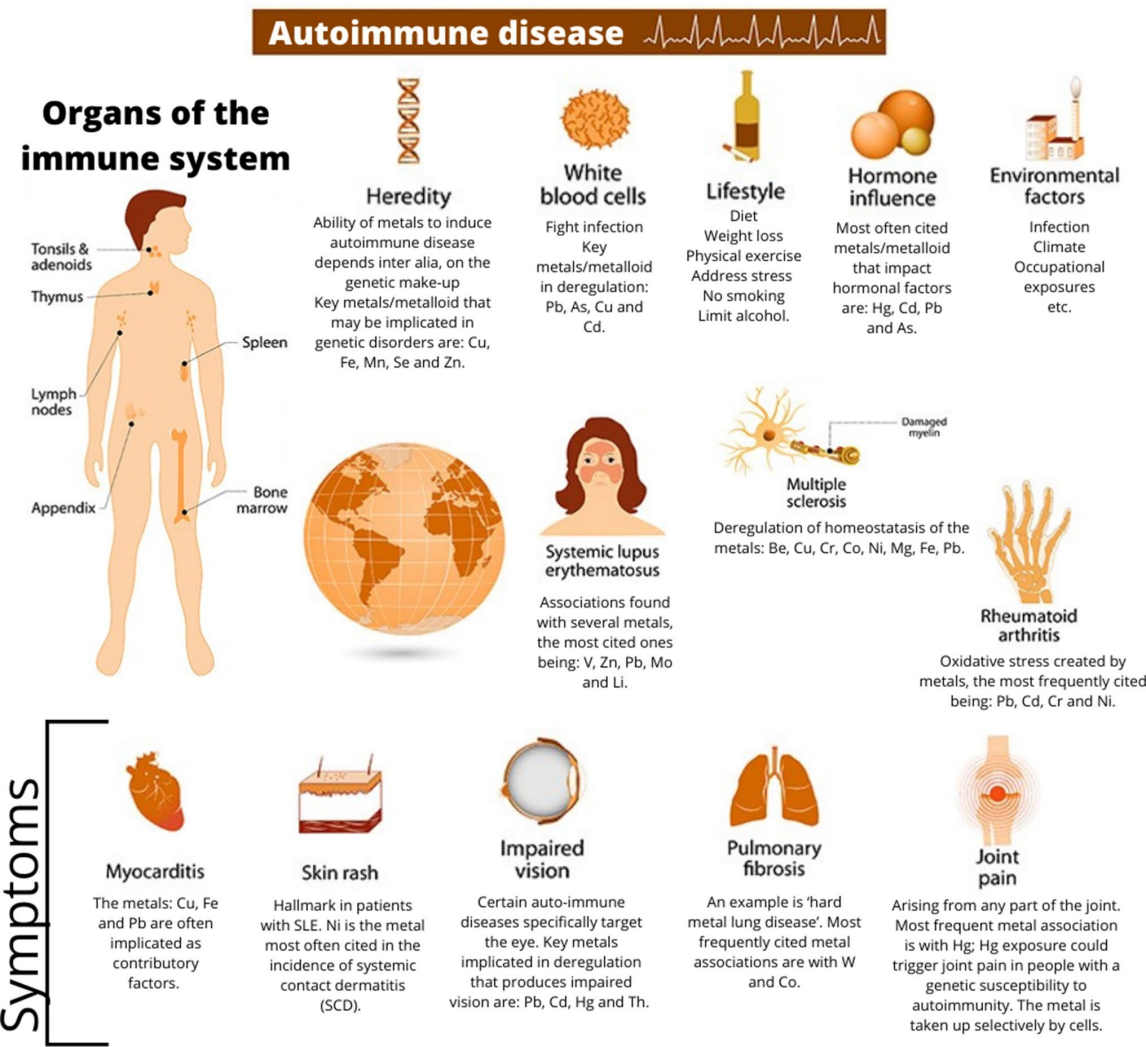
Schematic illustration of the link between trace element (particularly metal-) deregulation in organs of the immune system and development of autoimmune diseases. The main trace elements/metals involved in deregulation are given for each corresponding autoimmune disease portrayed. Image by the U.S. National Institute of Environmental Health Sciences

A number of studies (e.g., Balali-Mood et al. [284]; Liu et al. [285]; Singh et al. [60]) have found that excess levels of *ROS* produced as a result of bioaccumulation of metals during cellular events (disruption of metal ion homeostasis) lead to *oxidative stress*, which can overwhelm the body’s antioxidant protection, inducing DNA damage. These events can promote the development of certain *metabolic* diseases whose precise aetiologies are still unknown, e.g., Type 1 diabetes [122].

The state of oxidative stress is characterised by an imbalance between production and accumulation of ROS in cells and tissues and the ability of living systems to detoxify these reactive products. An excellent review written by Jomova and Valko in 2011 [286] illustrates how redox active metals such as Fe, Cu, Cr, Co and others undergo redox cycling reactions and have the potential of producing reactive radicals such as superoxide anion radical and nitric oxide in biological systems.

Other conditions associated with oxidative DNA damage (genetic) include neurodegenerative disorders of unknown aetiology such as AD and PD (Coppedè and Migliore [287]; Singh et al. [60]), autoimmune diseases such as rheumatoid arthritis, systemic *Lupus erythematosus* (SLE) and many others (Ramani et al. [288]).

### Role of climate change

The relationship between climate change and health is becoming increasingly clear and well documented. Developments in this area of research can be followed up in a number of recent publications (e.g., Grobusch and Grobusch [289]; Romanello et al. [290]; WHO [291]; and many scientific journals are devoted exclusively to this subject or have whole sections addressing it. *With DUA, specifically, however, the relationship with climate is much less clear and relatively few studies or publications exist on the subject.*

Attempts in grappling with the challenges of global climate change have revealed unexpected findings on immune system mediation by toxic trace elements, infectious disease (re)emergence, and the growing field of epigenetics (See, e.g., Ackland et al. [292]). These findings have helped us recast the environment as an agent of illness. Reflecting this shift, leading international bodies assessing the science related to climate change, such as the WHO and the Intergovernmental Panel on Climate Change (IPCC) [e.g., through its Fifth Assessment Report], respectively, have begun to focus attention on contingent, non-linear, and cross-scalar cause and effect relationships between the environment and human health ([293, 294]).

## Geochemical variables and disease

There are a number of geochemical variables that determine the behaviour of trace elements/metals/metalloids before and after entry into biological systems. Before entry, parameters such as speciation, and those of the physical environment (e.g., pH and salinity) are among the key determinative factors. Upon entry into the body, parameters such as the dose, the chemical form, bioavailability, bioaccessibility and efficacy of transport mechanisms within the body become crucial. These factors must be considered in relation to host factors, such as age, gender, size and genetic characteristics; and in the case of the built environment, socio-economic conditions (e.g., the quality of the living space), and risk perception. Some of these factors enhance uptake and absorption, whereas others moderate it. It is thus possible to postulate that detailed measurement and thorough understanding of these variables can help us better chart the aetiology of DUA.

### Speciation

Changes in speciation take place as trace elements/metals/metalloids migrate within and between the environmental compartments of air, soil, water, sediments and biota. The fate of the different species of trace elements/metals/metalloids during these processes is controlled by key biogeochemical parameters including: pH (solubility), Eh, ionic strength (activity and charge-shielding), and dissolved organic carbon (complexation). A knowledge of speciation is therefore important in working out transport mechanisms, mode of accumulation, bioavailability and, in the context of the DUA, their toxicity and potential as diagnostic aid.

### Use of variations in natural isotopic composition in tissues for DUA diagnosis and/or prosnostic

The importance of variations in natural isotopic compositions, which, like metal concentrations, might provide useful clues in the unravelment of DUA causality, must never be overlooked. The natural abundance of heavy stable isotopes such as ^13^C, ^15^ N and ^18^O varies between tissues and metabolites due to isotope fractionation effects in biological processes. Indeed, as recently pointed out by Hastuti et al. [295], variations in stable isotope ratios of essential elements can reflect alterations in their homeostasis resulting from physiological changes in malignancies with higher sensitivity than concentrations do.

Such discrimination between heavy and light isotopic forms, alongside alterations in metabolic fluxes, takes place during enzyme or transporter activities, and may reflect metabolic deregulations associated with many DUA (Tea et al. [118]). *However, there is a paucity of research on causes of isotope fractionations in critical metabolic processes; and hence, we have little understanding to date of the mechanisms by which the isotopic signature of diseases are imprinted.*

Moynier et al. [296] observed that the isotopic composition of copper and zinc in AD brains differs from that of controls in a way that is statistically significant. Copper, with its multiple redox states (Cu^+^ and Cu^2+^), its isotopic fractionation is enhanced by redox change, which apparently, could explain the larger and statistically more significant isotopic shift observed for copper relative to zinc. In a previous article, Moynier et al. [297] stated that the connection between zinc and brain aging makes it possible to use changes in zinc homeostasis in AD to chart the evolutionary course of the disease. Sauzéat et al. [298] also revealed that copper and zinc isotopic compositions in CSFs (cerebrospinal fluids) of patients with ALS (amyotrophic lateral sclerosis) and AD, age-matched controls show that isotopic measurements of copper in CSF may provide a more credible understanding of the ALS disease than elemental concentrations do, and holds the potential to buttress existing information regarding the mechanisms involved in the development of ALS.

Tea et al. [118] provides a synopsis on current state of knowledge on changes in natural isotope composition in various tissue samples such as hair, plasma and saliva of patients compared to controls, discuss the metabolic origin of such isotope fractionations and reviews the prospect of using natural isotopic abundances for medical diagnosis and/or prognostic.

### Bioavailability

Bioavailability, from the standpoint of disease development, refers to the extent and rate at which an essential nutrient (e.g., nutritional element, vitamin, protein, water) enters systemic circulation and becomes available at the site of metabolic action. Bioavailability tends to be very variable and depends on such factors as age, sex, genetic phenotype and physical activity. *Low bioavailability of the active moiety such as a metabolite means that the amount absorbed by the body is too low for maintaining vital reactions; thus, presenting clues for the unravelment of certain DUA, inter alia.*

## Geochemical variables and the immune system

Numerous geoenvironmental factors can modulate human immunity; and it is necessary to understand their interactions in development of the immune system. Such an understanding enables us to address specific aspects of diseases, such as in unravelling the aetiology of DUA; but also, to identify methodological pathways to follow in our bid to determine the necessities for attaining long-term, life-long protection from disease. Here, an attempt is made to amalgamate existing data into a cohesive vision that illustrates how exposure to geoenvironmental variables, more especially, the geochemical-, can leave a lasting impression on the human immune system, and how this impression can either have beneficial or potentially deleterious effects.

### Cells of the immune system

The immune system is a complex network of cells and proteins that finds and attacks infectious agents such as bacteria, viruses and fungi (Nicholson [299]). The three broad categories of immune system cells are: *lymphocytes* (T-cells, B-cells and NK cells), which are a type of white blood cells; *neutrophils*, and *monocytes/macrophages*. Each cell type has specialised functions. For instance, *neutrophils* are important in fighting bacteria and fungi, while *lymphocytes* generally fight viruses. The distribution of metal- and metalloid species within a cell or tissue type, referred to as the *metallome*, constitutes an important study in the context of DUA.

### Principles of infection and immunity

According to Galask et al. [300], virtually any organism may behave as a pathogen under the right set of conditions; and therefore, it is more instructive to place organisms along a continuum from lesser to greater virulence, rather than classifying them as either pathogens or nonpathogens. Galask et al. [300] also contend that: “… among individual human hosts, there is a continuum in the intrinsic ability of each host to resist infection.”

As long ago as 1934, Theobald Smith suggested, in what is now, perhaps the most insightful statement of the relationship between microbial virulence and host resistance to infection, that: disease was a function of the number of organisms with which a host is initially infected multiplied by the virulence of the organism [301]. This relationship is considered to accurately reflect the nature of the infectious process today, despite modern changes in the ecology of infections.

Smith’s equation states:

1 $${\text{Disease}} = \frac{{{\text{Number of organisms}} \times {\text{Virulence of organisms}}}}{{{\text{Hosts resistance to infection}}}}$$

One can see from Eq. 1 that the result of a host’s encounter with an infectious agent, even a proven pathogen, will not necessarily be an infectious disease. However, if the host’s immunity becomes lowered for some reason, or if the host becomes overwhelmed by an increasing number of organisms, disease may appear, even with an organism of relatively low virulence. Another noteworthy point about Eq. 1, is its practical significance, which contributes to the clinician’s knowledge about the role of the individual host in infectious disease (Galask et al. [300]).

There are numerous mechanisms by which *trace elements/metals/metalloids* are absorbed, distributed, modified and stored in the body, and subsequently eliminated. Only a very brief look at immune system mechanisms is presented here, and only with reference to its interactions with trace elements, including metals and metalloids. Readers interested in further details should consult the many excellent publications on the topic (such as: Failla [302]; Keen et al. [303]; Plumlee and Ziegler [304]; Plumlee et al. [305]; Galask et al. [300]; Chaplin [306]; Winans et al. [307]; Nicholson [299]; Marshall et al. [308] and Paludan et al. [309].

*Toll-like receptors* (TLRs) which are located either on cell surfaces or within *endosomes* (See: “Glossary of Terms”, this article), are type I integral transmembrane receptors involved in the recognition and conveyance of pathogens (including trace elements/metals/metalloids) to the immune system (El-Zayat et al. [310]). Some micronutrients (vitamins and trace elements) may be considered as important TLR regulators, as they have immunomodulatory functions. Vitamins D, B12 and A, zinc, copper and iron, for instance, have important roles on innate immune responses (El-Zayat et al. [310]).

Thurnham’s 2004 review [311] summarises work on, *inter alia*, “… interactions between nutrients and genes, the influence of gene polymorphisms on micronutrients, the impact of immune responses on micronutrients and specific interactions of antioxidant micronutrients in disease processes to minimise potential pro-oxidant damage.” Mineral deficiency-induced abnormalities in the immune system are particularly profound when they occur during early development (Failla [302]).

In addition to the effect of trace elements on immune function, several studies have shown that, at certain levels, some of these elements, such as selenium can influence the genetics of a viral pathogen (Ermakov and Jovanović [312]). Thus, trace element nutrition influences not only the host response to a pathogen but also the pathogen itself (See e.g., Beck [313]).

Factors that influence the toxicities of substances that encounter the body in *bioaccessible* form (those that are readily released from Earth materials into the body fluids) include: *the exposure route, the dose, the chemical form of the substance at exposure, and the processes that chemically transform the substance during absorption, transport and metabolism* (Plumlee et al. [305]; Finkelman et al. [314]; Hasan [315]). *Sometimes, immanent errors of trace element metabolism occur to produce disease, such as when there are basic defects in the trace element transport mechanism* (See e.g., Danks [316]; Ferreira and Ghal [317].

### Immunotoxicity due to metals

In 2015, Nriagu and Skaar [318] noted that many countries experiencing infectious diseases endemia also have the highest prevalence of trace metal deficiencies or increased rates of trace metal pollution in the air, soil and water environments. These authors also pointed out the increased human susceptibility resulting from adverse effects of metal exposure (at suboptimal or toxic levels), and vice versa*, *viz., that metal excess or deficiency can increase the incidence or severity of infectious diseases.

Metals and metalloids influence the function of immunocompetent cells by a variety of mechanisms. Several of these metals and metalloids are known to be *immunotoxic*, including: aluminium, arsenic, beryllium, cadmium, cobalt, chromium, copper, iron, mercury, magnesium, manganese, nickel, lead, selenium, tin, vanadium and zinc. Depending on the particular metal, its speciation, concentration and bioavailability, and a number of other interdependent (geomedical) factors, a continuous metal/metalloid exposure will result in an *immunosuppression* or *immunoenhancement effect* (Kakuschke and Prange [319]).

According to Cabassi [320]), immunotoxicity occurs: “… either direct action of the free metal on the cell membrane or other organs of immunocytic components or by catalysis or inhibition of numerous enzyme reactions that are essential to cellular metabolism”. These interactions interfere with expression of the immune response. In this connection, Cabassi [320] notes that immunopotentiating effects are observed with certain metals when they occur at low concentration levels, whereas at high concentration levels, immunosuppression is the result. Theron et al. [321] affirmed this observation and went on to point out that it holds true particularly for toxic metals such as cadmium, mercury and lead, due to their cytotoxic effects which induce *apoptosis* and/or *necrosis* of immune cells leading to diminished effectiveness of the immune defences to infection.

Cabassi [320] describes some of the immunosuppression effects earlier identified by Descotes [322] that xenobiotics (including trace elements, metals and metalloids) can produce, such as “… changes in leucocyte cellularity, lymphocyte sub-population, reduced resistance of the organism to immune specific alterations, immunosuppression with increased susceptibility to infection and tumour development, immunostimulation with hypersensibility and development of autoimmune diseases.”

### Autoimmune diseases

*Autoimmune diseases* (AuDs) are a heterogeneous group of chronic conditions that affect specific target organs or multiple organ systems. These diseases occur when the body’s immune system functions abnormally, mistakingly attacking and destroying healthy body tissues, or causing abnormal organ development, or changes in organ function. Over 80 types of autoimmune disorders are known.

Among the different environmental factors that are known to influence the development of AuDs are: infections, low vitamin D levels, UV radiation, and *melatonin* [323, 324], which factors are also known to exhibit seasonal variation patterns that could influence disease development, severity and progression. Autoimmune disorders may cause destruction of body tissues,

In 2004, Descotes [322] recapitulated on the importance of autoimmunity as an important area of immunotoxicology, especially because autoimmune diseases affect a significant proportion of the world population, and some GETTS (*Genetic* testing Evidence Tracking Tool) experimental data suggest the existence of a possible association between *chemical exposures* and autoimmunity. There are literally thousands of chemicals and xenobiotics that we know can modulate the immune system (See e.g., Vojdani and Vojdani, [325]), *but we know very little about their specific effects on this system, and whether they may lead to autoimmunity*.

For metals, in particular, we know that there are several factors that determine the ease with which they can induce autoimmune disease—these include heredity (genetic makeup) (Fig. 3), speciation, dose, route of exposure, overall health, age and gender (See, e.g., Zhang and Lawrence [326]). *However, the precise mechanism by which this happens is still far from clear (Rowley and Monestia* [327]; *Bolon* [328].

Many questions remain as to how pathogenic challenge may interfere with immune system regulation and give rise to autoimmunity; and it is likely that other apparently unexplored immune modulatory mechanisms (*e.g., trace element/metal interaction*) also contribute to clinical AuDs (Fig. 3. *But, till quite recently, the exact etiopathogenesis of AuDs is still not well-defined* (See, e.g., Getts et al. [329]).

## Criticality of the ‘optimal range of intake’ and the occurrence of nutrient toxicities

Writing on the criticality of the optimal range for the micronutrient elements, Mao et al. [2] observed that this should correspond to an intake level of dietary requirement for an essential trace element that meets a specified criterion for adequacy, thus minimising or obviating the risk of nutrient deficiency or excess. The development of pathologic states and diseases will be the obvious result, should disruption in trace element homeostasis occur.

Many nutrients have an antagonistic relationship to one another, which can mean that when one is too high, it causes the other to become too low; and this could increase one’s susceptibility to infectious disease which may be acute or chronic. No pair of elements better exemplify this relationship than copper and zinc, which is as a result of their complex interactions in metabolic processes. In children and adults, the normal copper/zinc (Cu/Zn) ratio is about 1:1 (Faber et al. [330]; Bjørklund [82]). A similar ratio 1.0 ± 0.3 is given for body fluids (e,g., plasma) of healthy adults (Bahi et al. [331]; Kazi Tani et al. [332]).

There are many imponderables, though, that can bring about imbalances, chief of which, is the type of diet (Böckerman et al. [333]. A high intake of copper may adversely affect the absorption or utilisation of zinc, and vice versa. In other words, when your Cu/Zn ratio becomes out of balance, many health problems can occur, such as various neurodegenerative diseases (e.g., Büchl et al. [334]).

Excesses or deficiencies of trace elements/metals/metalloids and infectious diseases often co-occur and are the result of complex metabolic interactions. Most of our essential nutrient intake is from our diet, though thankfully, this portion alone is unlikely to bear excessive element intake levels. However, the consumption of fortified foods or supplements can also raise the level of trace elements/metals/metalloids and hence increase the chance of toxicity.

Environmental or occupational exposure to potentially toxic levels of elements/metals/metalloids induce concentrations that are bioavailable to immune cells, high enough to affect their function. Such an imbalance of the immune system caused by pollutants may play a significant role in the incidence of infectious diseases (See e.g., Erickson et al. [335]; Osredkar and Sustar [336]; Hara et al. [20]). In any case, our bodies have an elaborate system for managing and regulating the amount of key trace elements and limiting or eliminating the potentially toxic elements (PTEs) circulating in blood and stored in cells (Osredkar and Sustar [336]). It is when this system fails to function correctly that metabolic disturbances occur, with abnormal levels and ratios of trace elements/metals/metalloids developing and paving the way for occurrence of infectious disease (See e.g., Chandra [337]; Chaturvedi et al. [338]).

The concept of *nutritional immunity* in the context of host defense against pathogens (Djoko et al. [339] perceives a role for mechanisms by which a host organism sequesters trace elements/metals/metalloids to limit invading pathogens during infection. *Calprotectin*, for example, can restrict the acquisition of zinc or manganese (Kehl-Fie et al. [340]). The question remains however, as to whether the host is able to exploit the toxic properties of transition metal ions and use them as bactericides? (See Djoko et al. [339]).

## Disease risk mapping and dua cluster detection

According to Lahr and Kooistra [341] the value of risk maps lies in assisting analysts and scientists characterise the spatial nature of the effects of environmental stressors such as pollutants (e.g., arsenic, mercury, lead and chromium). Environmental risk maps are used as a means for conveying the results of complex environmental risk assessments to public health authorities, policy makers, urban planners, and other stakeholders in the general public.

### Cluster analysis and mapping of DUA

We know that diseases often occur in *clusters* (See, e.g., Whitty and Watt [342]). This is so, because of a common risk factor. Earlier, in 2016, Rodo et al. [343] reviewed the relevance of environmental factors to health outcomes of ailments whose causes are still poorly understood (*cf*., DUA). These authors listed several examples of emerging diseases belonging to this category, and surprisingly sharing some common epidemiological features such as their appearance in clusters (grouped geographically; and temporarily progress in nonrandom sequences that repeat year by year in a similar way). Rodo et al. [343] also noted that these diseases exhibit concurrent trend changes within regions in countries and among different world regions. Their list included: rheumatic diseases such as vasculitides, some inflammatory diseases, or even severe childhood acquired heart diseases, KD (Kawasaki disease), Henoch-Schönlein purpura, Takayasu’s aortitis, and anti-neutrophil cytoplasmic antibody (ANCA)-associated vasculitis.

It is important to map these clusters and to decipher which of them are non-random, since this can help us, *inter alia*, in unearthing new mechanisms for disease, which, in turn, can lead to the charting of aetiologies (*cf.*, DUA).

### Geochemical mapping

In 2014, Pinto et al. [344] described geochemical mapping as the base knowledge needed for delineating the regions of the planet with critical contents of PTEs from either natural or anthropogenic sources. These authors went on to identify sediments, soils and waters as the vehicles which link the inorganic environment to life through the provision of essential macro- and micronutrients; and that the chemical composition of surficial geological materials may bring about metabolic changes leading to the occurrence of endemic diseases in humans.

In the above context, it is possible to suggest that, for us to create a better understanding of the relationship between surficial geochemistry and public health (*cf*., DUA) it is necessary, first, to construct complete geochemical maps at appropriate scales across national boundaries, depicting the surficial distribution of all non-gaseous chemical elements (See: Darnley et al. [344]). The construction of such detailed maps of element distribution depicting regions of high levels of toxic compounds or those depleted in essential elements, is an urgent requirement for the proper assessment of the geochemical milieu regarding DUA causation.

Such maps have already been drawn for China [(See: Wang et al. [345]; Xie et al. [346]; Cheng et al. [347], England and Wales (See: Rawlins et al. [348], Australia (See Reimann and de Caritat [349]) the USA (See: Smith et al. [350]), and a few other countries]. An overlay of epidemiological maps (of disease distribution) on these geochemical maps would make possible the depiction of areas where disease clusters overlie anomalous element distribution (in water or soil), and so permit an evidence-based statistical assessment of the magnitude of any geochemical component in the disease causative web.

## Conclusions

This paper has advanced reasons why greater consideration should be given to co-factors linked to the geoenvironmental milieu, especially geochemical variables, in understanding causality of DUA. The medical profession, environmental health practitioners and allied scientists, relevant government officials and other stakeholders are made aware of the huge potential contribution of medical geologists and environmental geochemists in teams investigating the causes of DUA and sudden disease outbreaks. The following additional conclusions are drawn and some directions for future research, highlighted.

(I) There is currently an increasing worldwide trend in environmental geochemistry research towards determining the circulation of both nutritional elements and PTEs in the water-soil-food crop nexus, that enter the food chain. The prime motivator of this approach is considered to be the increasing concern about the significance of the entry—largely through the diet—of varying concentration levels of elements that may be bioavailable for negative interactions in metabolic processes that produce diseases, some of whose diagnoses are still ill-defined (*cf*., the DUA).
(II) The redox activity of metal ions can generate highly reactive species that impair DNA, giving rise to different oxidation products, the types and nature of impairment depending upon a number of factors, of which, the redox potentials of the DNA bases, formation of intermediate adducts, and identity of the reactive species are, perhaps, the more important of these factors (See: Angelé-Martínez et al. [351].
(III) It thus seems probable that improved knowledge on the influence of metal ion binding on the DNA structure and the differing binding behaviour of various metal ions could prove critical in elucidating the aetiology of a number of DUA in the future. As pointed out by Hegde et al. in 2011 [126] we can have a scenario in which a possible aetiological linkage exists between defects in BER/SSBR (See: “Glossary of Terms”, this article) and certain DUA, viz., the neurodegenerative diseases, as well as the restorative potential of metal chelators for DNA repair capacity.
(IV) The human immune system is complex, with numerous environmental factors modulating it early in life. As such, the system is constantly in a state of flux, trying to adapt to various local constraints and conditions imposed by selective pressures of our environment. This inherent plasticity means that our exposure to different geochemicals (metals, metalloids, radionuclides and transuraniums) and pathogenic organisms can result in undesirable outcomes (*cf*., DUA).
(V) After decades of research on the complexity and developmental trajectory of the foetal-neonatal immune system (See e.g., Amarasekera et al. [352]; Jain [353]; Scanlon [354], we are only just beginning to acquire knowledge and insights on the participation of trace elements/metals/metalloids in the selection, maturation and early activation events of the immune cells. Judicious use of modern analytical tools in cell biology- and molecular genetics research, and array technology, will no doubt hasten our understanding of outcomes in these metabolic processes. The position of the “metallome” in deciphering unknown aetiologies such as in the case of SIDS and that of many other DUA needs urgent research!
(VI) A functional immune system able to prevent or limit infections of the host, is particularly important for many rural populations where exposure to novel infectious occurs frequently. From the evidence adduced in this article, it is becoming increasingly clear that the amount of trace elements/metals/metalloids taken up largely through the diet, and its outcome in metabolic processes (leading either to accumulation or to deficiency in human tissues), has a significant control on whether the exerted effects are toxic or beneficial. As we gradually begin to fully understand these processes, food safety regulators will have the important and urgent task of re-considering, harmonising and updating current legislative regimes regarding the concentrations of trace elements/metal/ metalloids in food and in drinking water.
(VII) In order to promote immune-mediated health for life, we must consider the importance of our exposure to geoenvironmental variables and the dynamics of pathogen invasion in immune programming. To do this, however, we still need to seek knowledge on several aspects of immune system programming that starts in early life, and its influence on the risk of developing various DUA. Such research would generate information needed for articulation of future public health initiatives and for drawing renewed attention to the vulnerability of children in early life.
(VIII) Only recently (2021), Tea et al. [118] brought our awareness to the realisation that in many human diseases, including DUA, the natural abundance of stable isotopes in affected tissues might provide additional information helpful to better constrain and diagnose them. We still do not know enough about what causes isotope fractionations in specific metabolic reactions; and hence, do not fully understand the precise mechanisms at the origin of the isotopic signature of diseases. More basic research on both metabolic fluxes and enzymatic isotope effects is therefore necessary to increase the possibility of discovering new diagnostic biomarkers based on stable isotopes.
(IX) It is submitted that the efficiency of cluster investigation teams would be greatly enhanced by inclusion of medical geologists and environmental geochemists, from whom information on significant geoenvironmental exposure/exposure to geochemicals can be obtained, as well as for an increased potential for unravelment of environment and disease relationships. Whenever sudden disease outbreaks appear in clusters, it is always desirable to examine changes in the ambient soil, water and air trace element/metal/metalloid composition for any association with the disease. The overarching need for development of techniques for recognising the grouping of cases of a particular disorder in space and time (disease clusters), is that this may provide useful clues about the underlying aetiology (of DUA).
(X) It is submitted that the construction of correlation maps featuring complete geochemical databases, would, among other applications, enable the depiction of areas where disease clusters overlie anomalous element distribution (in water, soil or air), and so permit an evidence-based statistical assessment of the magnitude of any geochemical component in the disease causative web.

## Glossary of terms

- *Acute disease/illness* is any disease or illness that develops quickly, is intense or severe and lasts a relatively short period of time, or, any condition, e.g., infection, trauma, fracture—with a short (often less than 1 month) clinical course.
- *Amyloids* are aggregates of proteins characterised by a fibrillar morphology of 7—13 nm in diameter, a beta sheet secondary structure and ability to be stained by particular dyes. Amyloidosis is a rare disease that occurs when an abnormal protein, called *amyloid,* builds up in your organs and interferes with their normal function.
- *Apoptosis* refers to an orderly process of cell breakdown that occurs in multicellular organisms.
- *BER* refers to ‘base excision repair’ which is the main pathway for repair of base lesions, which is known to be associated with DNA replication.
- *Bias: In the field of statistics, bias* refers to the tendency of a *statistic* to overestimate or underestimate a parameter.
- *Calprotectin* is a protein biomarker released by a *neutrophil* when there is inflammation in the gastrointestinal (GI) tract, resulting in an increased level in the stool.
- *Chakra* (pl. *chakras*), a concept is found in the early traditions of Hinduism, refers to various focal points used in a variety of ancient meditation practices, collectively denominated as Tantra, or the esoteric or inner traditions of Hinduism. [Wikipedia, 2021. https://en.wikipedia.org/wiki/Chakra (accessed 20.01.2021)].
- A *chronic condition* is a human health condition or disease that is persistent or otherwise long-lasting in its effects or a disease that comes with time. The term chronic is often applied when the course of the disease lasts for more than three months.
- *Communicable diseases* are those that can be spread from person to person via an infectious agent, such as bacteria, viruses, fungi or parasites. *Non-communicable diseases* (NCDs) are the conditions or diseases which are not caused by transmission of infections like that in communicable diseases.
- A *confounding factor* also called a *confounding variable*, or *confounder* is a third variable in a study examining a potential cause-and-effect relationship. A *confounding variable* is related to both the supposed cause and the supposed effect of the study.
- *Correlational research* is a type of non-experimental research method in which a researcher measures two variables, understands and assesses the statistical relationship between them with no influence from any extraneous variable. “*Correlation* is not *causation*” means that just because two things *correlate does not* necessarily *mean* that one causes the other.
- *Endosomes* are membrane-bound vesicles, formed via a complex family of processes collectively known as endocytosis, and found in the cytoplasm of virtually every animal cell.
- *The etheric body, ether*-*body* or *æther body*, is a name given by neo-Theosophy to a vital *body* or subtle *body* coined by esoteric philosophers to describe the first or lowest layer in the “human energy field” or *aura*. It is thought to be in immediate contact with the physical *body*, to sustain it and connect it with “higher” *bodies*.
- *Gametes*, also referred to as *sex cells*, are an organism’s reproductive cells.
- *Homeostasis*. In biology, the tendency towards a relatively stable state (equilibrium)—internal, physical, and chemical conditions—maintained in physiological processes while adjusting to changing external conditions. *Dyshomeostasis*, on the other hand, refers to an imbalance or other breakdown of a homeostasis system.
- *Infectious diseases* are disorders caused by organisms such as bacteria, viruses, fungi or parasites. Many organisms live in and on our bodies. They are normally harmless or even helpful. But under certain conditions, some organisms may cause disease.
- The *immune system* is a series of complex defence mechanisms found in humans and other vertebrates, that helps to combat and destroy pathogenic organisms such as bacteria, fungi, viruses, and parasites. The immune system consists of two types of response mechanisms: (i) An *antigen-specific adaptive immune response mechanism,* also referred as the *acquired immune system*, which is composed of specialised, systemic cells and processes that eliminate pathogens by preventing their growth; and (ii) The *innate immune system* is a collection of cells and proteins that are functionally diverse and that defend against invasion by foreign organisms. An *innate immune response mechanism*, also called *natural*, is the set of processes that operate to protect the host from the surrounding environment in.
- *Immunosuppression* refers a state of decreased immunity.
- *Lymphocytes* are white blood cells that are also one of the body’s main types of immune cells.
- *Macrophages* are large, specialised cells that detect, engulf and destroy bacteria and other harmful organisms.
- *Melatonin* (sometimes referred to as the sleep hormone) is a natural hormone made by the *pineal* gland (a pea-sized gland situated just above the middle of the brain). It plays a central role in the body’s sleep–wake cycle. With its production rising with evening darkness, it promotes healthy sleep and helps orient our *circadian rhythm* (natural internal processes that follow a 24-h cycle).
- *Meridian* (as used in acupuncture and Chinese medicine) refers to each of a set of pathways in the body along which vital energy is said to flow.
- *Metallome:* In biochemistry, the *metallome* is the distribution of metal ions in a cellular compartment.
- *The miasma theory* (also called the *miasmatic theory*) is one in the field of medicine proffering that that certain diseases were caused by a *miasma* (μίασμα, Ancient Greek for “pollution”), form of bad air, quite noxious, and also known as night air.
- *Mitochondrial dysfunction* occurs when the mitochondria (tiny compartments that are present in almost every cell of the body) fail to work correctly, due to another disease or condition.
- *Monocytes* are the largest type of leukocyte (white blood cells). As a part of the vertebrate innate immune system monocytes also influence the process of adaptive immunity.
- * Mutations* are permanent changes in the DNA sequence, and they are a main cause of diversity among organisms.
- * Myalgia*: Pain in a muscle or group of muscles.
- * Necrosis* refers to the premature death of cells in living tissue when too little blood flows to them as a result of disease or injury.
- *Neutrophils* are a type of white blood cell. Most of the white blood cells that lead the immune system’s response are neutrophils.
- *Neurodegenerative disorders* are illnesses that involve the death of certain parts of the brain.
- An *oligomer* is a molecule consisting of a few similar or identical repeating units which could be derived, actually or conceptually, from copies of a smaller molecule, its monomer.
- *Pathogenesis* refers to the way (biological mechanism) in which a disease develops. *Pathogenicity* is the ability of an agent to cause disease (i.e., to harm the host).
- In genetics, a *promoter* is a sequence of DNA (deoxyribonucleic acid) to which proteins bind that initiate transcription of a single RNA (ribonucleic acid) from the DNA downstream of it. [Wikipedia, 2021. Promoter (genetics). https://en.wikipedia.org/wiki/Promoter_(genetics) (accessed 26.01.2021].
- *Reactive oxygen species* (ROS): An unstable molecule that contains oxygen and that easily reacts with other molecules in a cell. ROS are the contributors of oxidative stress which leads to various diseases and disorders.
- *Shank proteins* are multidomain scaffold proteins of the postsynaptic density that connect neurotransmitter receptors, ion channels, and other membrane proteins to various metabolic pathways.
- *SSBR* refers to single-strand breaks in DNA, which are discontinuities in one strand of the DNA double helix.
- SIDS is the abbreviation for ‘sudden infant death syndrome’, also known as ‘cot death’ or ‘crib death’, which is the sudden, unexpected and unexplained death, usually during sleep, of a seemingly healthy child of less than one year of age.
- A *syndrome* is a set of medical signs and symptoms which are correlated with each other and often associated with a particular disease or disorder. [Wikipedia, 2020. Syndrome. https://en.wikipedia.org/wiki/Syndrome (accessed 10.01.2021)].
- *Toll-like receptors* (TLRs) are a class of proteins (receptors) that constitute the first line of defence system against microbes.
- “A *xenobiotic* is a chemical substance found within an organism that is not naturally produced or expected to be present within the organism. It can also cover substances that are present in much higher concentrations than are usual.” [Wikipedia, 2020. https://en.wikipedia.org/wiki/Xenobiotic (accessed 26.01. 2021)].

## Data Availability

No data pertaining to human tissues were reproduced from articles reviewed in this study, obviating the need for ethical approval.
